# Two pediatric patients with hemiplegic migraine presenting as acute encephalopathy: case reports and a literature review

**DOI:** 10.3389/fped.2023.1214837

**Published:** 2023-07-28

**Authors:** Yu Xiang, Fei Li, Zhenfeng Song, Zhi Yi, Chengqing Yang, Jiao Xue, Ying Zhang

**Affiliations:** Department of Pediatric Neurology, The Affiliated Hospital of Qingdao University, Qingdao, China

**Keywords:** pediatric, hemiplegic migraine, encephalopathy, diagnosis, treatment

## Abstract

**Introduction:**

Hemiplegic migraine (HM) is a rare subtype of migraine. HM in children may be atypical in the initial stage of the disease, which could easily lead to misdiagnosis.

**Methods:**

We report two cases of atypical hemiplegic migraine that onset as an acute encephalopathy. And a comprehensive search was performed using PubMed, Web of Science, and Scopus. We selected only papers that reported complete clinical information about the patients with CACNA1A or ATP1A2 gene mutation.

**Results:**

Patient #1 showed a de novo mutation, c.674C>A (p. Pro225His), in exon 5 of the CACNA1A gene. And patient #2 showed a missense mutation (c.2143G>A, p. Gly715Arg) in exon 16 of the ATP1A2. Together with our two cases, a total of 160 patients (73 CACNA1A and 87 ATP1A2) were collected and summarized finally.

**Discussion:**

Acute encephalopathy is the main manifestation of severe attacks of HM in children, which adds to the difficulty of diagnosis. Physicians should consider HM in the differential diagnosis of patients presenting with somnolence, coma, or convulsion without structural, epileptic, infectious, or inflammatory explanation. When similar clinical cases appear, gene detection is particularly important, which is conducive to early diagnosis and treatment. Early recognition and treatment of the disease can help improve the prognosis.

## Introduction

Hemiplegic migraine (HM) is a rare subtype of migraine characterized by episodes of severe migraine and aura symptoms involving motor weakness or numbness, usually affecting one side of the body (hemiparesis), as well as visual, sensory, or speech disturbances (1). Severe attacks can be accompanied by seizures, coma, encephalopathy, fever, cerebellar involvement, cerebral edema, or cerebral infarction (2) and are often misdiagnosed as having postictal confusion, Todd’s paresis, and diagnosed as having epilepsy or viral encephalitis.

We report two cases of atypical hemiplegic migraine that started as an acute encephalopathy. The atypical clinical presentation of these two patients led to a challenging diagnosis. Therefore, we are reporting it to deepen the understanding of this disease. The goal is to reduce misdiagnosis, give patients appropriate and effective treatment, and improve the quality of their survival.

## Materials and methods

Blood samples (2 ml) were collected from patients and their parents. DNA was isolated from peripheral blood using a DNA Isolation Kit (Blood DNA Kit V2, CW2553). Concentrations were determined on a Qubit fluorometer (Invitrogen, Q33216) using a Qubit dsDNA HS Assay Kit (Invitrogen, Q32851). Agarose gel (1%) electrophoresis was performed for quality control. DNA libraries were prepared with a KAPA Library Preparation Kit (Kapa Biosystems, KR0453) following the manufacturer's instructions. Hybridization of pooled libraries to the capture probes (IDT xGen Exome Research Panel v1.0) and removal of non-hybridized library molecules were carried out according to the SeqCap Hybrid Mix System. Sample dilution, flow cell loading, and sequencing were performed according to the Illumina specifications. DNA libraries were sequenced on the Illumina NovaSeq platform as paired-end 200-bp reads.

Sequence variants were annotated using population and literature databases, including 1000 Genomes (http://www.1000genomes.org/), dbSNP (https://www.ncbi.nlm.nih.gov/snp/), GnomAD (http://gnomad.broadinstitute.org/), ClinVar (https://www.ncbi.nlm.nih.gov/clinvar/), HGMD (http://www.hgmd.cf.ac.uk/ac/index.php), and Online Mendelian Inheritance in Man (OMIM) (http://omim.org/). The variant interpretation was performed according to the American College of Medical Genetics (ACMG) guidelines.

## Case presentation

### Case report 1

Patient #1, a 3-year-old boy, was the second child of his unrelated parents. He was born via a normal pregnancy and delivery. His mother has a history of migraine. He was admitted to the Affiliated Hospital of Qingdao University because of headache, somnolence, status epilepticus, and fever for half a day.

**Figure 1:**
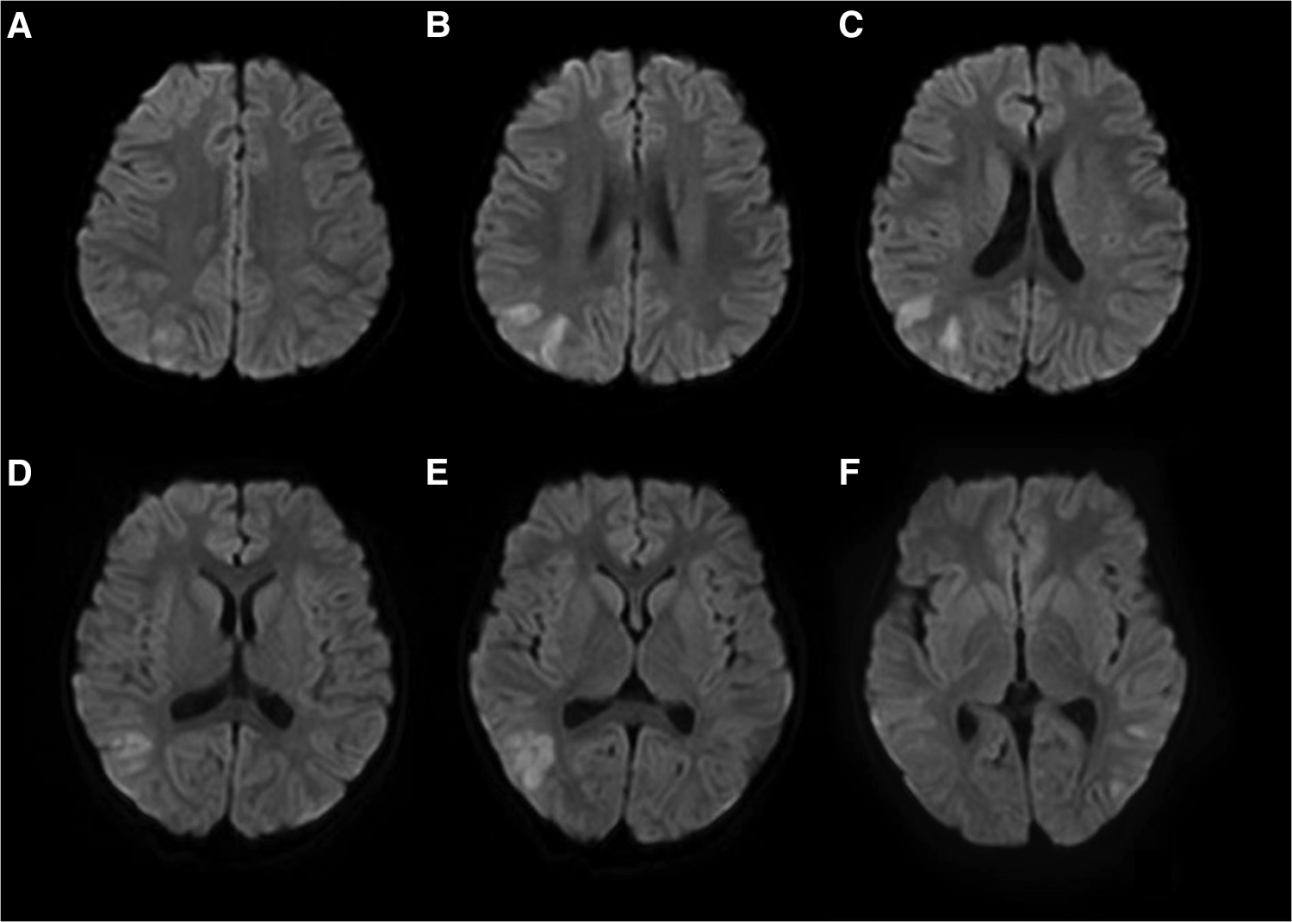
Brain MRI of patient #1 showed multiple high signal intensities on DWI in the right frontotemporal cortex (**A**–**F**).

The patient experienced a total of three similar episodes since the age of 1 year and 7 months. The patient's mental and motor development was normal before and after the onset of the disease. Each time, there was a minor head trauma, followed by headache, somnolence, and seizures. Each seizure manifested as a double-eye gaze to the left, intermittent left-sided limb rigidity, and unconsciousness. Each seizure manifested as status epilepticus. Half a day after the resolution of this seizure, the patient developed a fever with a temperature of 39°C. There were no abnormalities in brain magnetic resonance imaging (MRI), magnetic resonance angiography (MRA), and magnetic resonance venography (MRV) during previous episodes. The blood routine, arterial blood gas analysis, blood ammonia, glucose, homocysteine, calcium, and electrolytes showed no significant abnormalities. A brain MRI after this episode showed multiple high signal intensities on diffusion-weighted imaging (DWI) in the right frontotemporal cortex (Figure 1). Neurological examination after this episode showed the patient had left-sided central facial palsy as well as left-sided upper and lower extremity paralysis. Babinski's sign was positive bilaterally, the rest of the pathological symptoms were negative, and the meningeal stimulation sign was negative.

**Figure 2:**
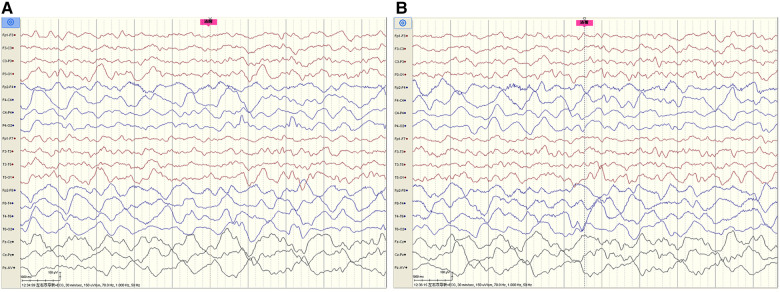
Video EEG monitoring of patient #1 showed persistent asymmetry between the right and left hemispheres and slow and sharp-slow wave emission in the right hemisphere. The absence of physiological waves in the right hemisphere is shown in **A**, **B**. The fuchsia rectangle above **A**, **B** indicates that Patient #1 is beginning to wake up.

**Figure 3:**
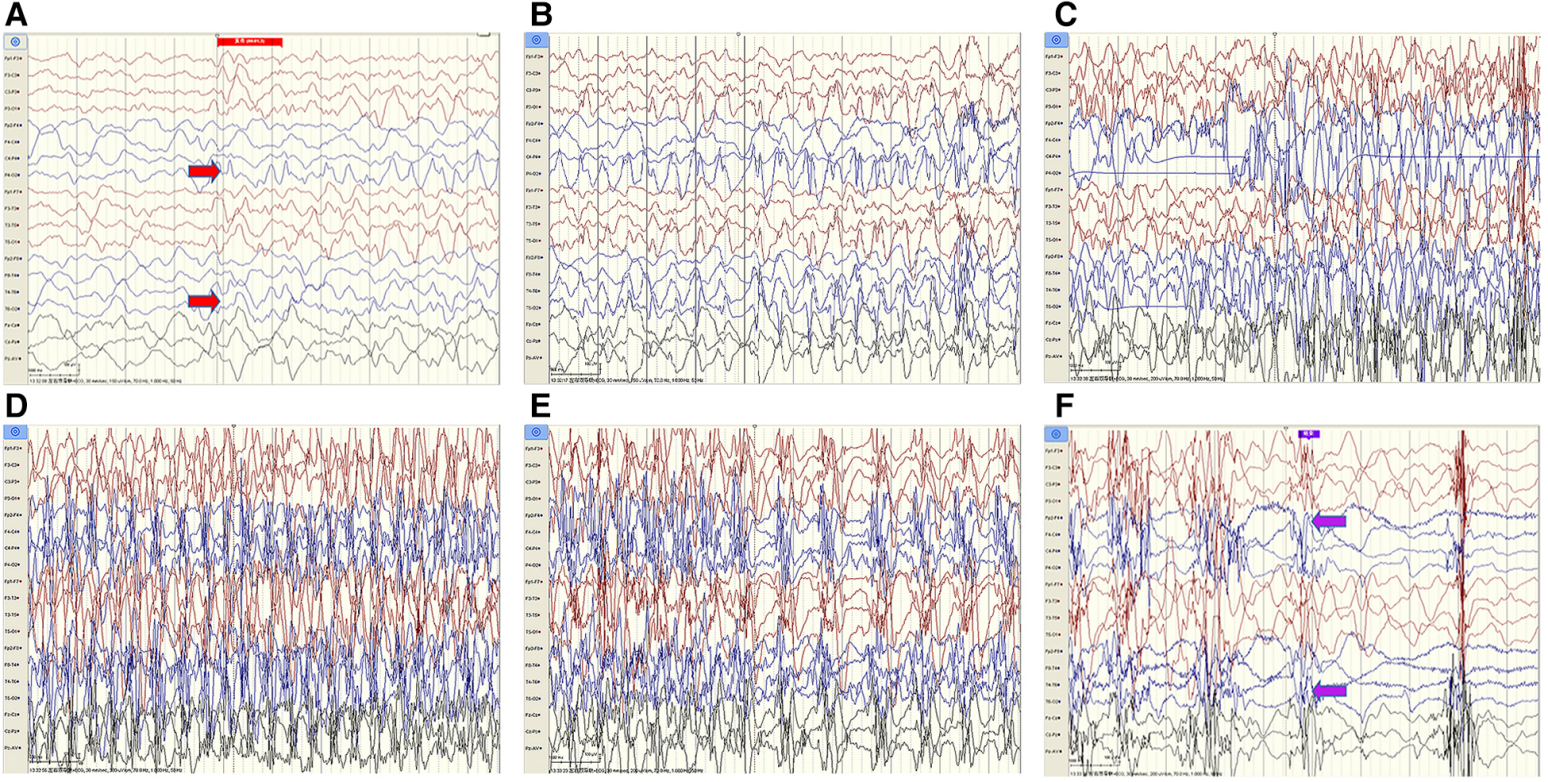
Patient #1 captured a focal seizure during video-EEG monitoring (**A**–**F**). Seizures started at the red arrow and stopped at the purple arrow for a total duration of about 90 seconds.

The cerebrospinal fluid (CSF) examination revealed that the pressure, glucose, chloride, protein level, cell count, and classification were all normal. The bacteria and fungi of CSF were negative. The peripheral blood and CSF demyelinating associated antibodies (MOG, AQP4, MBP) were all negative. Video electroencephalogram (EEG) monitoring showed persistent asymmetry between the right and left hemispheres and slow and sharp slow-wave emission in the right hemisphere. The absence of physiological waves in the right hemisphere is shown in Figure 2. During the same period, a focal seizure was monitored which lasted about 90 s (Figure 3).

**Figure 4:**
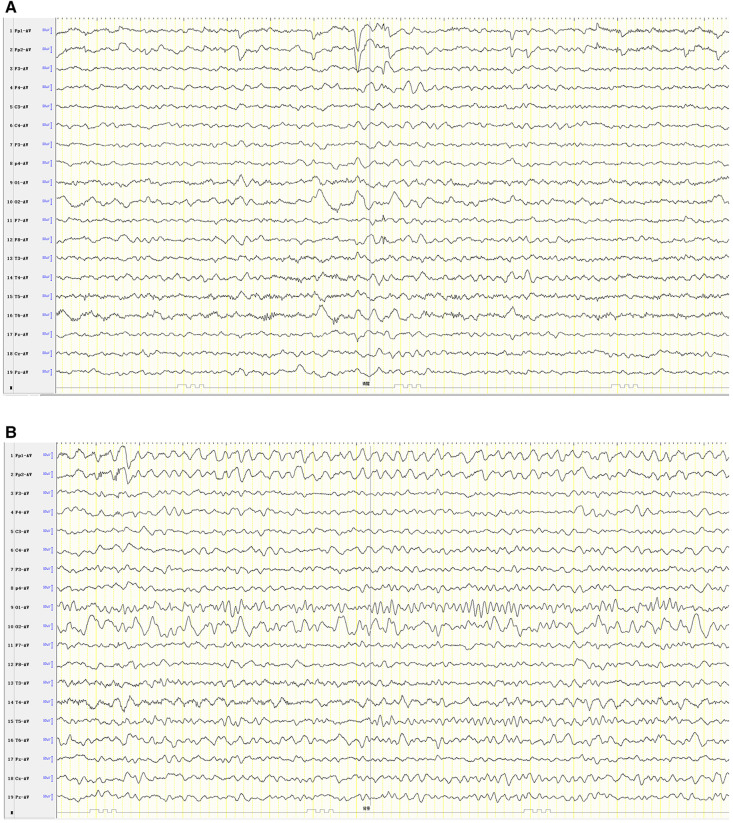
Patient #1 was reexamined for video EEG monitoring on day 11 of illness (**A**, **B**).

Combining the clinical manifestations of the patient's three attacks, we considered that the patient had a high probability of HM presented as an acute encephalopathy. In addition to conventional fluid and nutritional support therapy, the patient was given methylprednisolone (3 mg/kg/d) to reduce cerebral edema and mannitol (2.6 g/kg/d) to lower cranial pressure. Flunarizine (0.2 mg/kg/d) was given to dilate cerebral blood vessels. Disturbances in the patient's consciousness were ameliorated gradually. Left hemiplegia also gradually resolved, and he recovered completely without sequelae 9 days after the onset. The patient's review of video EEG monitoring on day 11 showed asymmetric background activity in the right and left occipital regions, with slow-wave coverage on the right side and predominantly slow and sharp slow-wave emission in the right hemisphere (Figure 4).

**Figure 5:**
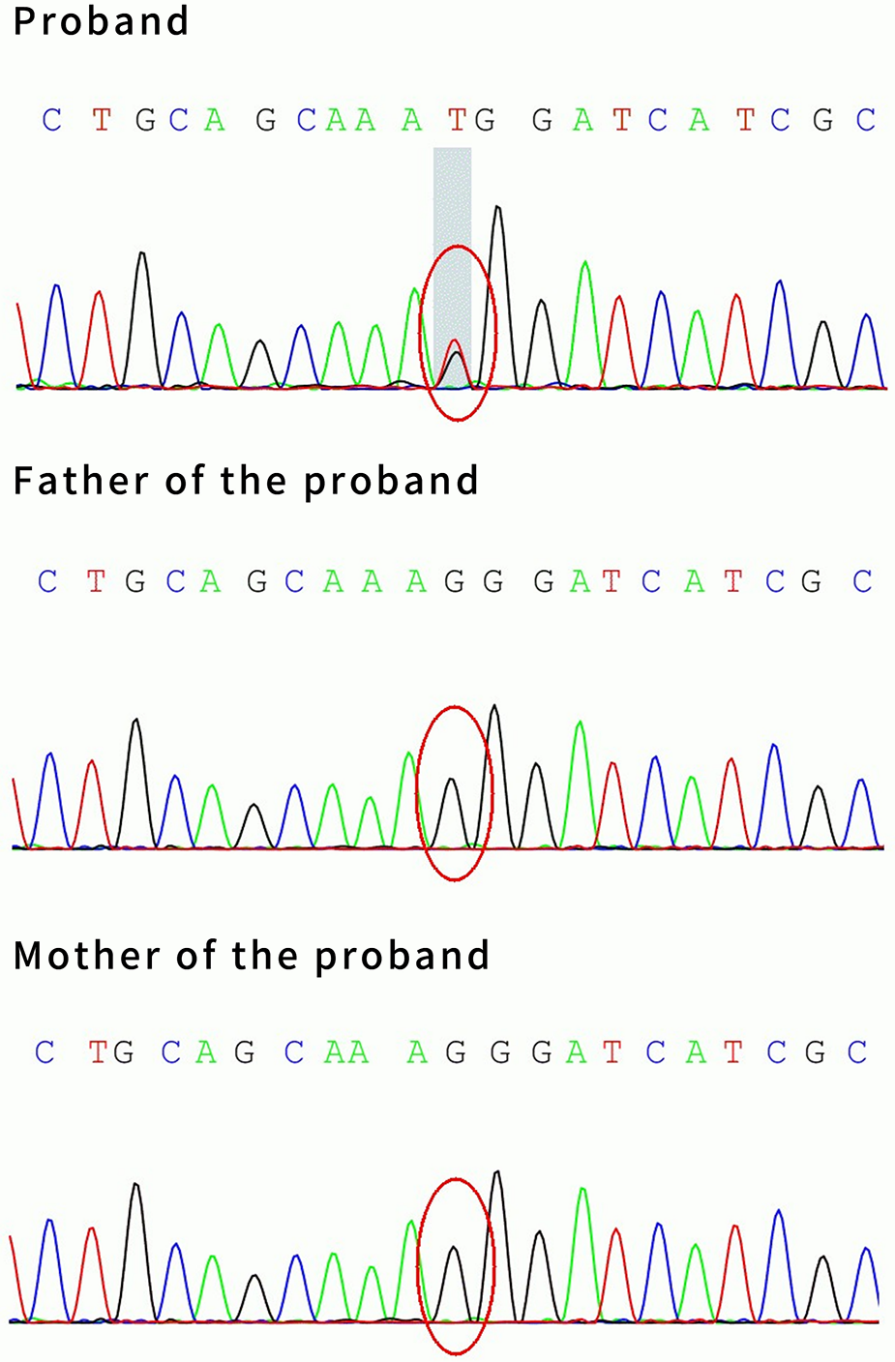
Patient #1 and his parents’ whole-exome sequencing results. Patient #1 has a de nove mutation of c.674C>A (p. Pro225His), in exon 5 of the *CACNA1A* gene. Neither of his parents carried the disease-modifying mutation. The circles indicate the locus of variation.

After communicating with the patient's family, whole-exome sequencing (WES) was performed in the Running Gene Medical laboratory in Beijing, China. WES found a *de novo* mutation, c.674C>A (p. Pro225His), in exon 5 of the *CACNA1A* gene (Figure 5). Neither of his parents had the mutation. This mutation was predicted to be disease-causing by Mutation Taster (http://www.mutationtaster.org/) and was predicted to be damaging with a score of 1.000 (sensitivity: 0.00; specificity: 1.00) by Polyphen 2 (http://genetics.bwh.harvard.edu/pph2/) and damaging with a score of 0.000 by SIFT (cutoff = 0.05) (http://sift.jcvi.org/www/SIFT_BLink_submit.html).

**Figure 6:**
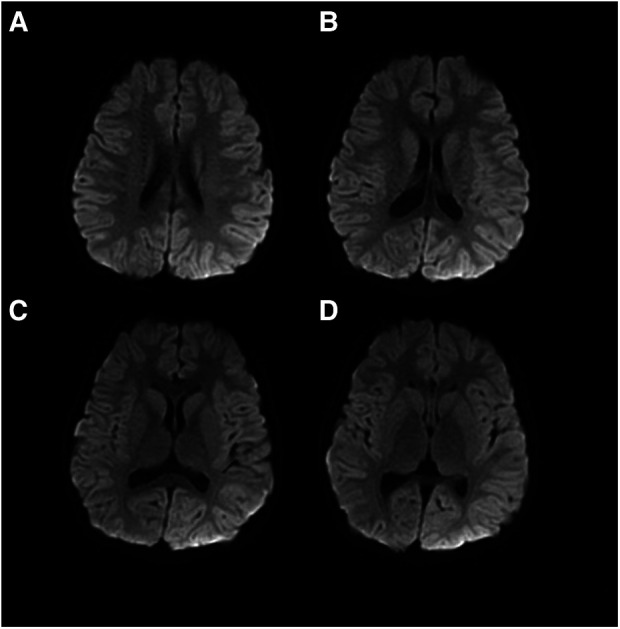
The brain MRI of Patient #1 showed complete disappearance of abnormal signals 1.5 months after treatment (**A**–**D**).

The patient was finally diagnosed with hemiplegic migraine presenting as an acute encephalopathy. He was given daily flunarizine 3 mg (0.2 mg/kg) to prevent HM attacks. After treatment for 1.5 months, a follow-up MRI showed complete disappearance of abnormal signals throughout the right hemispheric cortex (Figure 6). The video EEG was not reviewed because the patient fully recovered to the pre-onset level and the patient's compliance was not high.

### Case report 2

Patient #2 was a 5-year-old boy who was the second child of his unrelated parents. He was admitted to the Affiliated Hospital of Qingdao University because of focal seizures and somnolence for half a day. The seizure was characterized by double-eye gaze, cyanosis of the lips, and shaking upper limbs, which lasted for about 1 min. The body temperature was 38°C. The blood routine, blood electrolytes, ammonia, glucose, and calcium were all normal. Brain CT showed no significant abnormalities. Neurological examination showed a Glasgow Coma Scale of 8. The patient's extremity muscle strength and muscle tone were normal. Babinski's sign was positive bilaterally, while the rest of the pathological signs were negative, and the meningeal stimulation sign was negative.

The patient was born via a normal pregnancy and delivery. He presented with previously regular intellectual and motor development. He started to have febrile seizures at the age of 1 year and had a total of three times by the age of 2 years. He had a history of viral encephalitis 3 years ago. He suffered a headache, nausea, and vomiting after mild head trauma 2 years ago. The result of the brain CT was normal. Finally, the symptoms were relieved after rest. His mother had a history of seizures. His sister was diagnosed with epilepsy and was being treated with valproic acid. She also had a history of seizures and coma after mild head trauma.

The examination was immediately completed after admission. Blood biochemistry, blood homocysteine, blood culture, and herpes simplex virus test were all normal. A brain MRI was not performed on admission because the patient was uncooperative. Considering the patient's family history, we recommended that the patient complete the WES, but the family declined.

The patient was given a combination of mannitol (2 g/kg/d), methylprednisolone (2 mg/kg/d), meropenem (60 mg/kg/d), acyclovir (27 mg/kg/d), and vitamin B6 (6 mg/kg/d). On the second day of admission, the patient had another seizure, which lasted about 4 min. The body temperature was 38.2°C. He was treated with midazolam and completed a lumbar puncture. The biochemical and cytologic examination of the CSF revealed normal glucose, chloride, protein level, cell count, and classification. The CSF tests for bacteria, viruses, and fungi were all negative. The patient was continued to be given an ice blanket and ice cap to lower the temperature and combination therapy. On the fourth day of admission, the patient's temperature gradually decreased to normal, and no further seizures occurred. His consciousness gradually improved with continued treatment as described above. A brain MRI was performed on the 13th day of admission, and the results were normal. On the 14th day of admission, the patient recovered to the pre-onset level, submitted whole-exome sequencing, and was discharged.

**Figure 7:**
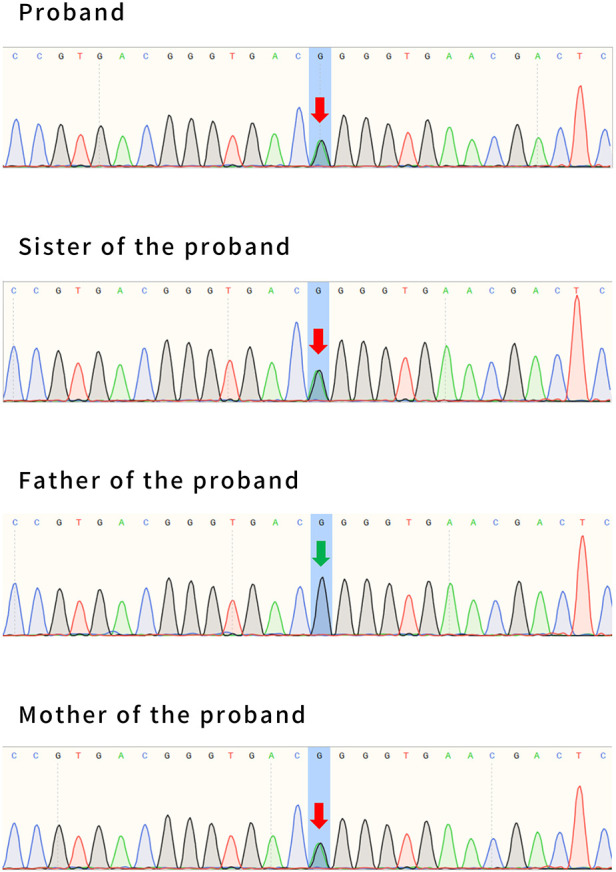
The whole-exome sequencing results of patient #2, his sister, and his parents. Patient #2 has a maternal mutation of c.2143G>A (p. Gly715Arg), in exon 16 of the *ATP1A2* gene. The same mutations were confirmed in his sister. His father had no mutation at this locus. The arrows indicate the locus of variation.

WES was performed by Fujun Genetics Medical Laboratory, Fuzhou, China. The result showed a missense mutation c.2143G>A (p. Gly715Arg) in exon 16 of the *ATP1A2* gene (Figure 7) previously reported as a pathogenic mutation (3, 4). The same mutations were confirmed in the patient's mother and sister (Figure 7). This mutation was predicted to be disease-causing by Mutation Taster (http://www.mutationtaster.org/) and was predicted to be damaging with a score of 1.000 (sensitivity: 0.00; specificity: 1.00) by Polyphen 2 (http://genetics.bwh.harvard.edu/pph2/) and damaging with a score of 0.000 by SIFT (cutoff = 0.05) (http://sift.jcvi.org/www/SIFT_BLink_submit.html).

Based on a series of examinations, the patient was finally diagnosed with acute encephalopathy of HM. He was subsequently treated with flunarizine at 5 mg/day.

## Discussion

The overall estimated prevalence of HM is 0.01% (5). The prevalence of sporadic HM (SHM) is at least 0.002% and the prevalence of familial HM (FHM) is at least 0.003% based on the Danish population (6–8). The onset is usually in the first or second decade (9). The frequency, intensity, and duration of HM attacks often decrease with age (5, 8). Emotional and intense physical stress, viral infections, and head trauma are the more commonly reported trigger factors for HM attacks (10). The two patients in this paper were both male and the age of onset was all before 5 years of age. For patient #1, the trigger was mild head trauma. The trigger for Patient #2 is unclear, and the child had runny nose before this episode, so we hypothesized that it might be related to a viral infection.

HM can be divided into FHM and SHM (11). If one first- or second-degree relative has similar symptoms, the disorder can be classified as FHM. Otherwise, it is classified as SHM (11). FHM can be classified as FHM1 (OMIM #141500), FHM2 (OMIM #602481), or FHM3 (OMIM #609634) according to mutations in *CACNA1A*, *ATP1A2*, or *SCN1A*, respectively (1, 5, 11). SHM can be caused by a *de novo* mutation in a gene that causes the familial form or by the inheritance of a gene mutation from an asymptomatic parent with FHM (12). A review study by Bonemazzi et al. found that pediatric SHM patients have longer and more severe attacks compared to FHM patients, especially during the initial years after disease onset, while FHM cases tend to have the disease for longer periods (8).

A comprehensive search was performed using PubMed, Web of Science, and Scopus. We selected only papers that reported complete clinical information about the patients with *CACNA1A* or *ATP1A2* gene mutation. We screened 97 articles finally. Together with our two cases, a total of 160 patients (73 CACNA1A and 87 ATP1A2) were identified and all features are summarized in Table 1.

**Table 1:** Clinical characteristics of HM patients with *CACNA1A* or *ATP1A2* gene mutation.

	*CACNA1A* (*n* (%))	*ATP1A2* (*n* (%))	References
Male	29/73 (39.7%)	45/87 (51.7%)	(2–4, 13–51)
Female	44/73 (60.3%)	42/87 (48.3%)	(14, 15, 17, 20, 23, 25, 31, 35, 37, 38, 41–43, 47, 50, 52–78)
Headache	62/73 (84.9%)	75/87 (86.2%)	(2–4, 13, 14, 16, 17, 19–21, 23–29, 31–39, 41–52, 55, 56, 59, 60, 63, 65–75, 77, 79)
Visual, sensory, speech/language symptoms	43/73 (58.9%)	63/87 (72.4%)	(2, 13–15, 17, 20, 25–27, 29–31, 33, 35–50, 52, 53, 55, 56, 58–61, 67–72, 74, 75, 77–79)
Motor weakness	12/73 (16.4%)	39/87 (44.8%)	(15, 16, 25, 26, 29, 30, 32, 33, 36–38, 41, 42, 44, 46, 47, 49, 50, 52, 59, 60, 63, 71, 72, 74, 75, 77, 78)
Fever	28/73 (38.3%)	26/87 (29.9%)	(2–4, 13, 14, 16, 17, 19, 21, 22, 25, 37, 39, 40, 43, 51, 52, 54, 55, 62, 64, 66, 67, 69, 71, 72, 74, 77, 79)
Hemiplegic	33/73 (45.2%)	46/87 (52.9%)	(2–4, 13, 14, 17, 19, 20, 22, 23, 25, 27–31, 33, 34, 36, 37, 39, 43, 45–48, 51–57, 61, 65–73, 75, 76, 78, 79)
Encephalopathy	31/73 (42.4%)	34/87 (39.1%)	(2, 3, 13, 14, 17, 18, 22, 25, 29–31, 34, 35, 37, 39–41, 43, 47, 49, 51–55, 57, 62, 64, 66, 67, 69, 71–76, 78, 79)
Seizures	25/73 (34.2%)	20/87 (22.9%)	(3, 17, 22, 23, 25, 28, 29, 34, 35, 37, 38, 40, 41, 43, 47, 52–54, 57, 62, 64, 68, 69, 72, 73, 78, 79)
Mental retardation/learning disability	38/73 (52.0%)	16/87 (18.4%)	(13–16, 18, 19, 22–26, 28–30, 34, 39–41, 43, 51–54, 56, 61, 62, 67, 73, 78, 79)
Cerebellar atrophy	36/73 (49.3%)	1/87 (1.1%)	(13–23, 26, 52–54, 56, 58, 62, 63, 65, 67, 69)
Ataxia or nystagmus	44/73 (60.3%)	5/87 (5.7%)	(2, 13–20, 23–27, 49, 50, 52–54, 56, 58, 61–65, 67, 69, 79)

We found that about half of them presented with a typical hemiplegic migraine attack, i.e., fully reversible visual, sensory, or speech symptoms or motor weakness. Motor symptoms are most frequently localized in hands, arms, feet, legs, tongue, face, and body (8). The progression of weakness is always gradual, requiring at least 5 min, and the irradiation of the symptom is usually unilateral configuring a hemiparesis in most cases (8). Duration is about 30 min to 24 h (mean duration 5 h) for motor aura (8). Of the two patients we reported, patient #1 presented with hemiparesis symptoms that lasted more than 24 h, which is inconsistent with the description of motor aura symptoms in previous reports. Both patients reported in this paper did not describe any other aura symptoms, such as sensory aura, visual aura, or aphasic aura, which may be related to the patient's young age and lack of expressive skills.

However, some patients started with other atypical symptoms, such as encephalopathy. Of the reported patients with hemiplegic migraine with *CACNA1A* mutations, 42.4% presented with encephalopathy. Among patients with *ATP1A2* mutations, 39.1% presented with encephalopathy. The specific manifestations of encephalopathy were impaired level of consciousness, fever, or seizures, all lasting for >24 h. Patients with mild encephalopathy have only an impaired level of consciousness, while patients with severe manifestations of encephalopathy may have fever and seizures. In this paper, we found that all patients presenting with fever showed signs of encephalopathy. Fever may be central (79). In our group, both probands had a sudden onset of fever without a clear history of infection, and neither antibiotic nor antipyretic was effective for them. The clinical manifestations began to improve after the body temperature returned to normal in both probands. Both our patients manifested focal seizures followed by generalized seizures, of which patient #1 started with status epilepticus in each case. The whole-exome sequencing of patient #1 showed a *de novo* missense mutation of the *CACNA1A* gene, c.674C>A (p. Pro825His). By reviewing the literature, only one report of the Pro225His mutation was identified (80). The patient was an 8-year-old female who was born prematurely, and presented with developmental delay, speech and spatial sense impairment, poor concentration, and epilepsy (80). The patient had at least four HM episodes presenting as headaches and seizures (80). The difference is that our patient #1 was born at full term and had normal cognitive and motor development. He had no previous seizures. Similarly, our patient presented this time with status epilepticus and hemiplegia. However, we are currently unable to clarify the clinical phenotype of these mutations due to the paucity of reports. The whole-exome sequencing of patient #2 showed a maternally inherited missense mutation of the ATP1A2 gene, c.2143G>A (p. Gly715Arg). By reviewing the literature, three patients have been reported thus far with the same mutations as patient #2. Together with our case, Table 2 summarizes the clinical features of these four patients. All four patients had their onset in childhood and their age of onset ranged from 1 to 6 years, with our patient #2 having the youngest age of onset. However, because all four patients started with acute encephalopathy, this led to different degrees of diagnostic delay. Patients may present with fever, somnolence, hemiparesis, seizures, or aphasia during the acute phase of encephalopathy. All three patients showed cerebral edema in the acute phase, except for our patient #2 for whom no brain MRI data were in the acute phase. Patients’ encephalopathy symptoms may resolve within a few weeks, with recovery in up to 10 weeks. Combined with the clinical presentation of these four patients, we hypothesized that this mutation may be associated with early and severe HM manifestations.

**Table 2:** Summary of HM caused by *ATP1A2*, c.2143G>A mutation.

	Our patient #2	Chen et al. (3)	Sanctis et al. (4)	Zhang et al. (29)
Age at onset	1 years old	3 years old	6 years old	3 years old
Age of diagnosis	5 years old	11 years old	6 years old	5 years old
Gender	Male	Male	Male	Male
Symptoms	Fever, seizures, somnolence, and right hemiplegia	Fever, vomiting, somnolence, hemiplegia, seizures, aphasia, headache, and cognitive impairment	Fever, nausea, somnolence, aphasia, and right hemiplegia	Headache, fever, seizures, somnolence, and hemiplegia
MRI presentation	Normal	Unilateral cortical edema	Diffuse cortical edema of the left hemisphere	Slight swelling of the cortex of the right cerebral hemisphere
Recovery duration	3 weeks	10 weeks	several weeks	4 weeks

The mechanism of HM accompanied by acute encephalopathy is still unknown. Toldo et al. reported an 8-year-old female who had a prolonged attack of sporadic HM; the first MRI was negative while the neuroradiological follow-up documented a progressive increase of the cortical swelling with mild hyperintensity on DWI, suggesting intracellular edema (81). Technetium-99m-ethyl cysteinate dimmer (99mTc-ECD) single photon emission computed tomography (SPET) (day 27), performed when the hemiplegia was almost completely resolved but aphasia persisted, showed a marked unilateral cerebral hypoperfusion. This finding suggested a primary neuronal dysfunction with reduced uptake of the radiopharmaceutical by “stunned neuronal cells” (81). The neuroimaging findings have been related to the possible underlying pathogenic mechanisms, ion channel dysfunction caused prolonged neuronal depolarization followed by the shift of water from the extra- to the intra-cell compartment and then cellular swelling and neuronal loss (81).

The outcome is favorable in most patients; however, neurological conditions and comorbidities can be associated (8). Some studies reported neurological signs in up to 60% of patients, with the most frequent neurological alterations including cerebellar ataxia, nystagmus, postural tremor, and clumsiness (8). The majority of FHM1 patients, often more than half of the families (2), and a small number of FHM2 patients had cerebellar signs, and cerebellar atrophy was seen on brain MRI (82). Cerebellar atrophy may become apparent with age (83). In our group, both patients experienced multiple hemiplegic migraine attacks, but neither of them showed cerebellar signs, cerebellar atrophy, motor development lag, or regression, but it cannot be excluded that this is related to the young age of both patients and long-term follow-up is needed. In addition, Wada et al. considered that the mutation of *CACNA*1*A* itself, rather than the frequency of coma attacks, may play a central role in the pathogenesis of progressive cerebellar atrophy (14). Cerebellar signs are rare in patients with FHM2. Three patients reported by Spadaro et al. showed cerebellar symptoms such as nystagmus, gait ataxia, dysmetria, and dysarthria, one of the patients also showed cerebellar atrophy (69). It was thought that the presentation may be related to the patient's alcohol or sedative intake (84). However, in other families, no other reasons for the cerebellar signs such as alcohol, drugs, CACNA1A or SCA mutations, or vascular brain disorders could be found, suggesting that these signs are part of the FHM2 phenotype in this family (69).

We found that about half of the HM patients with the *CACAN1A* gene mutation had mental retardation, which may be related to the calcium channel impairment caused by the *CACNA1A* gene mutation. For our patient #1 with *CACNA1A* gene mutation, he experienced three hemiplegic migraine attacks, and no mental retardation or regression manifestation has been found yet, which may be related to the patient's young age or insufficient follow-up time. Observation of this patient should continue to be strengthened in the future. Overall, more research is needed to explore the underlying mechanisms of acute encephalopathy in HM.

At present, the impact of the *ATP1A*2 mutation on cognitive profile in HM patients has not been evaluated in detail (40). In this paper, we found that most of the effects of the *ATP1A2* gene on intelligence were transient and could gradually return to pre-initiation levels after the remission of HM symptoms. However, Wang et al. reported a patient with cognitive dysfunction in a specific area (40). He had trouble in mathematics and depicting three-dimensional things. It is suggested that a patient with HM with *ATP1A2* could develop permanent cognitive dysfunction (40). As for our patient #2, a careful review of his history revealed that the patient and his sister were normal in all aspects except for poor numeracy. The patient's mother had cognitive impairment, but she refused to undergo intelligence testing. The cognitive function test is an issue we need to focus on when following up with patient #2 in the future.

Patients with HM need only symptomatic treatment in the acute phase to bring about symptom relief. Some reports indicated that a prompt recovery had been described in three patients with *CACNA1A* mutations through the combined use of steroids and hypertonic solutions in the course of encephalopathy and cerebral edema (54, 83, 85). For FHM2, NMDA receptor antagonist memantine could prevent glutamatergic excitotoxicity, and idebenone, dl-NBP, as well as traditional anti-migraine drugs could protect the mitochondria function (73). We report two patients who started with acute encephalopathy and were treated symptomatically in the acute phase. Both patients were given low-dose methylprednisolone combined with mannitol. No drugs such as memantine and idebenone were applied to patient #2 because the diagnosis had not been established before. The WES results can help us select more effective medication in the future.

Prophylactic management is applied to patients with frequent, long-lasting, or severe attacks (12). A single-center cohort study of pediatric migraine lasting 11 years suggested that flunarizine should be used as first-line medication in children with hemiplegic migraine (86). In the two patients we reported, considering the patients’ age, weight, and tolerance to the drug, the dose of flunarizine was 0.2 mg/kg per day, with a maximum amount of 5 mg/d. The two patients we reported were treated with flunarizine for 8and 2 months, respectively, and both were well tolerated without adverse effects. During the current follow-up, patient #1 had occasional headaches that could be resolved with rest. He has not suffered any head trauma and has not undergone severe attacks such as convulsions or encephalopathy. He is still under continued follow-up. Patient #2 did not experience any further headaches or convulsions, probably due to the short follow-up period, and is still under continued follow-up.

## Conclusions

Acute encephalopathy is the main manifestation of severe attacks of HM in children, which adds to the difficulty of diagnosis. Physicians should consider HM in the differential diagnosis of patients presenting with somnolence, coma, or convulsion without structural, epileptic, infectious, or inflammatory explanation. Early recognition and treatment of the disease can help improve the prognosis. Whole-exome sequencing can suggest diagnosis and the type. Future multicenter and large sample size studies are still needed to explore the treatment strategy for this disease.

## Data Availability

Original datasets are available in the NCBI repository: The original contributions presented in the study are publicly available. The BioProject ID is PRJNA993808. This data can be found here: http://www.ncbi.nlm.nih.gov/bioproject/993808.
